# Supplementary material for: Effect of gravity on colloidal particle transport in a saturated porous medium: Analytical solutions and experiments
Source: PLoS One. 2022 Oct 5;17(10):e0275644. doi: 10.1371/journal.pone.0275644 (PMC9534398; doi:10.1371/journal.pone.0275644)
Supplement: S1 Appendix — (DOCX) [file pone.0275644.s001.docx]

# Appendix A: Derivation of the Analytical Solution

The desired analytical solution is obtained by solving the problem described by the following integrodifferential equation and initial/boundary conditions:

|  | $\frac{\partial C(z,t)}{\partial t}=D\frac{\partial^{2}C(z,t)}{\partial z^{2}}-U_{\text{tot}}\frac{\partial C(z,t)}{\partial z}\text{ }+k_{de}\cdot k_{at}\cdot\int_{0}^{t} C(z,\tau)\cdot\exp\left[-k_{de}(t-\tau)\right]\text{d}\tau-k_{at}\cdot C(z,t)\text{ }$. | (A1) |
| --- | --- | --- |
|  | $C(z,0)=0$. | (A2) |
|  | $C(0,t)=g(t)$. | (A3) |
|  | $C(+\infty,t)=0$. | (A4) |

The Laplace transform is performed on *t* and *z*, and the transform variables are *s* and *r*, respectively. By combining the initial conditions $C(z,0)=0$ and the transformed boundary conditions $\bar{C}(0,s)=\bar{g}(s)$, the solution in the transformed domain can be obtained as:

| $\bar{C}(r,s)=\frac{\bar{g}\cdot r-\frac{U_{\text{tot}}}{D}\cdot\bar{g}+\frac{\text{d}\bar{C}(0,s)}{\text{d}z}}{(r+A)^{2}-B^{2}}$, | (A5) |
| --- | --- |

where $A=-U_{\text{tot}}/(2D),B=\left[A^{2}+\left(s+\frac{k_{at}\cdot s}{s+k_{de}}\right)/D\right]^{1/2}$.

The suspended matter concentration in the space-time domain is obtained by inverting the solution in the transform domain. First, the inverse Laplace transform for *r* is found, and the displacement property of the Laplace transform, the convolution theorem, and the Laplace transform table are utilized to obtain:

|  | $\begin{matrix} \bar{C}(z,s)=\frac{1}{B}\left[-\frac{U_{\text{tot}}}{D}\cdot\bar{g}+\frac{\text{d}\bar{C}(0,s)}{\text{d}z}\right]\cdot\exp(-Az)\cdot\sinh(Bz) \\ +\bar{g}\cdot\frac{\exp(-Az)}{B}\cdot[B\cdot\cosh(Bz)-A\sinh(Bz)] \end{matrix}$. | (A6) |
| --- | --- | --- |

By multiplying both sides of Equation (A6) by $2B\cdot\exp[(A-B)z]$ and applying the boundary conditions $C(+\infty,t)=0$, we obtain:

|  | $\frac{\text{d}\bar{C}(0,s)}{\text{d}z}=\frac{U_{\text{tot}}}{D}\cdot\bar{g}+(A-B)\cdot\bar{g}$. | (A7) |
| --- | --- | --- |

Equation (A7) is substituted into Equation (A6) to obtain:

|  | $\bar{C}(z,s)=\bar{g}\cdot\exp(-Az)\cdot\exp(-Bz)=\bar{g}\cdot\exp(-Az)\cdot h(z,s)$, | (A8) |
| --- | --- | --- |

where

|  | $h(z,s)=\exp(-Bz)=\exp\left[-\frac{z}{\sqrt{D\left(s+k_{de}\right)}}\left(s^{2}+s\left(k_{de}+E\right)+k_{de}E-k_{de}\cdot k_{\text{d}}\right)^{\frac{1}{2}}\right]$. | (A9) |
| --- | --- | --- |
|  | $E=DA^{2}+k_{at}$ | (A10) |

In the equation, it can be known from the literature [1] that:

|  | , | (A11) |
| --- | --- | --- |

where $L^{-1}$ is the inverse Laplace operator; $\overline{f_{0}}\left(s\right)$ is the Laplace transform of an arbitrary function $f_{0}(t)$; $\alpha$ is an arbitrary constant; and $I_{0}$ is a zero-order modified Bessel function of the first kind.

Suppose:

|  | . | (A12) |
| --- | --- | --- |

Accordingly, there are:

|  | $f_{0}(t)=\frac{\alpha_{1}}{\left(4\pi t^{3}\right)^{1/2}}\exp\left(-\frac{\alpha_{1}^{2}}{4t}-\alpha_{2}t\right)$, | (A13) |
| --- | --- | --- |
|  | $\begin{matrix} h(z,s)=\bar{f}_{0}\left(s+k_{de}-\frac{\alpha}{s+k_{de}}\right) \\ =\exp\left\{-\frac{\alpha_{1}}{\left(s+k_{de}\right)^{1/2}}\left[s^{2}+s\left(2k_{de}+\alpha_{2}\right)+k_{de}\left(k_{de}+\alpha_{2}\right)-\alpha\right]^{1/2}\right\} \end{matrix}$. | (A14) |

By comparing Equations (A9) and (A14), we obtain $\alpha=k_{de}k_{at},\alpha_{1}=z/\sqrt{D},\alpha_{2}=E-k_{de}$.

From Equation (A11), we obtain:

|  | $L^{-1}[h(z,s)]=L^{-1}\left[\frac{k_{de}\cdot h(z,s)}{s+k_{de}}+\frac{s\cdot h(z,s)}{s+k_{de}}\right]=k_{de}\cdot R(z,t)+\frac{\partial R(z,t)}{\partial t}$, | (A15) |
| --- | --- | --- |

where

|  | $R(z,t)=\exp(-k_{de}t)\int_{0}^{t} I_{0}\left[2(\alpha\eta(t-\eta))^{1/2}\right]\cdot\frac{\alpha_{1}}{2\sqrt{\pi\eta^{3}}}\cdot\exp\left[-\frac{\alpha_{1}^{2}}{4\eta}-\alpha_{2}\eta\right]\text{d}\eta$, | (A16) |
| --- | --- | --- |
|  | $\begin{matrix} \frac{\partial R(z,t)}{\partial t}=\exp(-k_{de}t)\int_{0}^{t} \left\{\left(\frac{\alpha\eta}{t-\eta}\right)^{1/2}\cdot I_{1}\left[2(\alpha\eta(t-\eta))^{1/2}\right]-k_{de}I_{0}\left[2(\alpha\eta(t-\eta))^{1/2}\right]\right\}\cdot\\ \frac{\alpha_{1}}{2\sqrt{\pi\eta^{3}}}\exp\left[-\frac{\alpha_{1}^{2}}{4\eta}-\alpha_{2}\eta\right]d\eta+\exp(-k_{de}t)\frac{\alpha_{1}}{2\sqrt{\pi t^{3}}}\exp\left[-\frac{\alpha_{1}^{2}}{4t}-\alpha_{2}t\right] \end{matrix}$. | (A17) |

Finally, by applying the convolution theorem to Equation (A8), the analytical solution of the suspended particle concentration is obtained:

|  | $C(z,t)=\exp\left(\frac{U_{\text{tot}}z}{2D}\right)\cdot\int_{0}^{t} g(\tau)\cdot\left[k_{de}\cdot W(z,t-\tau)+\frac{\partial W(z,t-\tau)}{\partial t}\right]\text{d}\tau$, | (A18) |
| --- | --- | --- |

where $\tau$ is a dummy variable and

|  | $W(z,t)=\exp(-k_{de}t)\int_{0}^{t} I_{0}\left[2(\alpha\eta(t-\eta))^{1/2}\right]\cdot\frac{z}{2\eta\sqrt{\pi D\eta}}\cdot\exp\left[-\frac{z^{2}}{4D\eta}-\frac{U_{\text{tot}}^{2}\eta}{4D}+\left(k_{de}-k_{at}\right)\eta\right]\text{d}\eta$, | (A19) |
| --- | --- | --- |
|  |  | (A20) |

**References**

1. Sim, Y., & Chrysikopoulos, C. V. (1998). Three-dimensional analytical models for virus transport in saturated porous media. Transport in porous media, 30(1), 87-112.
