# Supplementary material for: Effect of gravity on colloidal particle transport in a saturated porous medium: Analytical solutions and experiments
Source: PLoS One. 2022 Oct 5;17(10):e0275644. doi: 10.1371/journal.pone.0275644 (PMC9534398; doi:10.1371/journal.pone.0275644)
Supplement: Attachment — Submitted filename: Review-PONE-D-22-19180.pdf [file pone.0275644.s003.pdf]

Review of:

[Manuscript PONE-D-22-19180

Title: Effect of gravity on particle transport in a saturated porous medium: Analytical solutions and experiments

Authors: ---]

I have read the paper with interest.

The manuscript topic is of interest for "PLOS ONE".

The authors offer a work that potentially can improve our knowledge about the topic of interest. I suggest that the authors consider a revision of their work along with the following suggestions and questions.

Suggestions and Questions:

1- It is recommended to start the abstract with general information about the topic.

2- It is suggested to discuss more about the findings of this study in the abstract.

3- It is recommended to mention about the applications of this study at the end of abstract:
The findings of this study can help for better understanding of …

4- I strongly recommend the authors to add one paragraph discussing the difference between their work and the previously performed studies in literature. In other words, what is the novelty of this work? I offer the authors to revise the abstract and introduction in order to incorporate the novelty of their work. This change motivates the readers of "PLOS ONE" to study this work with interest.

5- It is suggested to add a figure in Section 1, which presents the general sketch of the problem under study.

6- It is recommended to include a paragraph at the end of introduction to present the steps of the work like:

First, the theoretical model is presented. Then,…

7- What are the advantages and disadvantages of this study? I recommend the authors to highlight this topic.

8- What are the limitations of this study? I recommend the authors to highlight this topic.

9- The quality of all the figures should be improved.

10- It is suggested to keep the main governing equations in the text of manuscript and move the rest of equations to an appendix.

11- Is it possible to show the steps of the work in terms of a flowchart?

12- The title for last section should be changed to “Summary and Conclusions”.

13- It is suggested to add a nomenclature (including alphabetic letters, Greek letters, subscripts, and superscripts).

14- The following relevant reference can be cited properly in introduction and throughout the manuscript:

- (2022), An extensive scope of flow loops with a focus on particle transport. Physics of Fluids, 34(8): 081301.
