# Supplementary material for: Effect of gravity on colloidal particle transport in a saturated porous medium: Analytical solutions and experiments
Source: PLoS One. 2022 Oct 5;17(10):e0275644. doi: 10.1371/journal.pone.0275644 (PMC9534398; doi:10.1371/journal.pone.0275644)
Supplement: Attachment — Submitted filename: Response to Reviewers.docx [file pone.0275644.s004.docx]

Dear Editors and Reviewers:

Thank you for your letter and for the reviewers’ comments concerning our manuscript entitled “Effect of gravity on particle transport in a saturated porous medium: Analytical solutions and experiments” (PONE-D-22-19180). Those comments are all valuable and very helpful for revising and improving our paper, as well as the important guiding significance to our researches. We have studied comments carefully and have made correction which we hope meet with approval. Revised portion are marked in red in the paper. The main corrections in the paper and the responds to the reviewer’s comments are as flowing:

**Responds to the reviewers’ comments:**

**Reviewer #1:**

1- It is recommended to start the abstract with general information about the topic.

Response: We appreciate it very much for this good suggestion. At the beginning of the abstract we added “The colloidal transport process in all porous media from laboratory to nature is affected by gravity”.

2- It is suggested to discuss more about the findings of this study in the abstract.

Response: We appreciate it very much for this good suggestion. We added the introduction of theoretical findings in the abstract, such as line 6 " In this paper, a mathematical model of colloidal particle migration in a saturated porous medium with the gravity effect is established by combining the gap velocity (advection) with the settling velocity (gravity effect), and an analytical solution of the particle migration problem with time variation of the particle injection intensity is obtained using an integral transformation.", line 11" The analytical solution can easily analyze the colloid transport experimental data in a variety of seepage directions".

3- It is recommended to mention about the applications of this study at the end of abstract:The findings of this study can help for better understanding of …

Response: At the end of abstract we added “The findings of this study can help for better understanding of colloidal transport properties in porous media under the coupled effects of gravity and hydrodynamics.”

4- I strongly recommend the authors to add one paragraph discussing the difference between their work and the previously performed studies in literature. In other words, what is the novelty of this work? I offer the authors to revise the abstract and introduction in order to incorporate the novelty of their work.

Response: We have added a paragraph in the introduction to discuss the differences between our work and previous studies conducted in the literature. The fourth paragraph“Although there are numerous mathematical models available that describe colloid transport in porous media, in this study, the frequently employed continuum approach was adopted. The phenomenological colloid transport model developed by Massei^[1^] was extended to account for colloid sedimentation, in order to examine how the flow direction influences colloid fate and transport in porous media. The conventional analytical solution based on a classical convection dispersion model^[1][2][3]^, in which the influence of the release effect on the deposition kinetics is not considered. Chen and Bai^[4]^ used an analytical solution considering the effect of dispersive flux on deposition kinetics (neglecting detachment) to simulate experimental results. These models are usually used to describe the breakthrough curves (BTCs) of column tests for the case of the so-called “pulse injection”, in which the duration of the injected pulse is sufficiently short to be considered instantaneous ^[1][3]^. However, a potential drawback of these theoretical models is that there is an increasing deviation of the model prediction from the experimental data as the injection time increases. It is very important to establish a solution considering the detachment effect of sorbed particles due to the long release process of particles for a sustained injection.”

5- It is suggested to add a figure in Section 1, which presents the general sketch of the problem under study.

Response: In Section 1, we added a schematic diagram of colloidal particles attached and suspended in porous space ( Figure 1).

Figure 1. Schematic of cplloids attachment and suspension

6- It is recommended to include a paragraph at the end of introduction to present the steps of the work like:

First, the theoretical model is presented. Then,…

Response: We appreciate it very much for this good suggestion, and we have done it according to your ideas. We introduced the steps in the last paragraph of the introduction, “First, the theoretical model is presented. Based on the classical particle migration model, this paper establishes a particle migration model in saturated porous media that considers the effect of gravity and obtains an analytical solution of particle migration when the particle injection intensity changes with time. Then, a glass bead-packed column experiment was conducted in three seepage directions (horizontal seepage, upward seepage, and downward seepage). The solution containing particles was injected at one end of a column, and the breakthrough curves of the particles were tested at the other end. Finally, A regression analysis was conducted on the test results to deeply study the effect the gravity mechanism on the particle migration characteristics in porous media.”

7- What are the advantages and disadvantages of this study? I recommend the authors to highlight this topic.

Response: The advantages of this study is that the theoretical model of colloidal transport takes into account not only the gravity effect but also the release effect on the deposition dynamics. We presented the disadvantages of the study and future work plans in the last paragraph of Section 5, “When the surface of porous media particles is irregular or rough, it is difficult to accurately calculate the particle settlement correction factor in the theoretical model. Therefore, smooth glass spheres are selected as the porous media in this paper, while the actual porous media surface is irregular or rough. Future studies should consider the role of gravitational force on colloid transport in saturated as well as unsaturated packed columns with irregular or coarse porous medium particles.”

8- What are the limitations of this study? I recommend the authors to highlight this topic.

Response: We presented the limitations of the study in the last paragraph of Section 2.1, “Considering that colloidal particles may not be able to penetrate porous media in extreme cases, the colloidal migration model proposed in this paper should have limited requirements for the particle size of porous media. Referring to literature [2] and [5], the particle size ratio of fine particles to porous media is limited to less than 0.1”

9- The quality of all the figures should be improved.

Response: We appreciate it very much for this good suggestion, and we have done it according to your ideas. We've improved the quality of all the pictures.

10- It is suggested to keep the main governing equations in the text of manuscript and move the rest of equations to an appendix.

Response: We have kept the main governing equations in the main text of the manuscript and moved the remaining equations to Appendix A: Derivation of the Analytical Solution.

11- Is it possible to show the steps of the work in terms of a flowchart?

Response: At the end of the first section, we used a flowchart to illustrate the steps of the work.

Figure 2. flowchart of the steps of the work

12- The title for last section should be changed to “Summary and Conclusions”.

Response: We appreciate it very much for this good suggestion, and we have done it according to your ideas. We have changed the title of the last section to "Summary and Conclusion".

13- It is suggested to add a nomenclature (including alphabetic letters, Greek letters, subscripts, and superscripts).

Response: We appreciate it very much for this good suggestion, and we have done it according to your ideas. We've added a nomenclature.

14- The following relevant reference can be cited properly in introduction and throughout the manuscript:

(2022), An extensive scope of flow loops with a focus on particle transport. Physics of Fluids, 34(8): 081301.

Response: We cited this literature appropriately in the second paragraph of the introduction, “In addition, Martin Brown^[6]^ found that Pipe inclination (horizontal to vertical) has great influence on particle transportation,and pipe with different deviations could lead to novel evaluations.”

Special thanks to you for your good comments.

**Reviewer #2:**

1- The authors should consider mentioning in the title that colloids are considered, or, almost equivalently, that the 1-D ADE including sorption is considered and solved

Response: We appreciate it very much for this good suggestion, and we have done it according to your ideas. We changed the title to “Effect of gravity on colloidal particle transport in a saturated porous medium: Analytical solutions and experiments”.

2- I would like to see in the revised version a clear indication of what is original and what was earlier achieved in the earlier works on the solution of the ADE with sorption that are mentioned in the introduction.

Response: We appreciate it very much for this good suggestion. We have added a paragraph in the introduction to discuss the differences between our work and previous studies conducted in the literature. The fourth paragraph“Although there are numerous mathematical models available that describe colloid transport in porous media, in this study, the frequently employed continuum approach was adopted. The phenomenological colloid transport model developed by Massei^[1^] was extended to account for colloid sedimentation, in order to examine how the flow direction influences colloid fate and transport in porous media. The conventional analytical solution based on a classical convection dispersion model^[1][2][3]^, in which the influence of the release effect on the deposition kinetics is not considered. Chen and Bai^[4]^ used an analytical solution considering the effect of dispersive flux on deposition kinetics (neglecting detachment) to simulate experimental results. These models are usually used to describe the breakthrough curves (BTCs) of column tests for the case of the so-called “pulse injection”, in which the duration of the injected pulse is sufficiently short to be considered instantaneous ^[1][3]^. However, a potential drawback of these theoretical models is that there is an increasing deviation of the model prediction from the experimental data as the injection time increases. It is very important to establish a solution considering the detachment effect of sorbed particles due to the long release process of particles for a sustained injection.”

3- The authors should consider rewriting their analytical solution and all relevant outputs in dimensionless form (this is easily achieved by finding adequate scales, and is now standard in this type of papers), and performing the comparison with experiments in such form.

Response: We appreciate it very much for this good suggestion. We changed the concentration *C* of the ordinate of the penetration curve in Section 4.1 (Figure 3) into the dimensionless relative concentration *C_R_* (*C_R_*=*C_out_V_P_*/*m*).

Figure 3. Particle penetration curves under the different seepage directions and different velocities

And we change effective particle velocity *U_tot_* in Section 4.2 (Figure 4) into the dimensionless (*U_tot_-U*)/*U.*

Figure 4. Relationship between the particle effective velocity and seepage velocity under three different seepage directions

Referring to the literature [2][3][4], the particle dispersion properties in Section 4.3 use the dispersion degree $\alpha_{d}$. The particle deposition characteristics in Section 4.4 use the adsorption coefficient *k_at_* and deposition rate *R*.

We tried to improve the grammar, syntax, English usage and the quality of all the figure.Special thanks to you for your good comments.

We tried our best to improve the manuscript and made some changes in the manuscript. These changes will not influence the content and framework of the paper. And here we did not list the changes but marked in red in revised paper.

We appreciate for Editors/Reviewers’ warm work earnestly, and hope that the correction will meet with approval.

Once again, thank you very much for your comments and suggestions

Reference:

[1] Massei, N., Lacroix, M., Wang, H. Q., & Dupont, J. P. (2002). Transport of particulate material and dissolved tracer in a highly permeable porous medium: comparison of the transfer parameters. Journal of Contaminant Hydrology, 57(1-2), 21-39.
[2] Bennacer, L., Ahfir, N. D., Bouanani, A., Alem, A., & Wang, H. (2013). Suspended particles transport and deposition in saturated granular porous medium: particle size effects. Transport in Porous Media, 100(3), 377-392.
[3] Ahfir, N. D., Wang, H. Q., Benamar, A., Alem, A., Massei, N., & Dupont, J. P. (2007). Transport and deposition of suspended particles in saturated porous media: hydrodynamic effect. Hydrogeology Journal, 15(4), 659-668.
[4] Chen, X., & Bai, B. (2015). Experimental investigation and modeling of particulate transportation and deposition in vertical and horizontal flows. Hydrogeology journal, 23(2), 365-375.
[5] Zhang, F., Wang, T., Liu, F., Peng, M., Bate, B., & Wang, P. (2022). Hydro-mechanical coupled analysis of near-wellbore fines migration from unconsolidated reservoirs. Acta Geotechnica, 1-17.
[6] Martin Brown, N., & Dejam, M. (2022). An extensive scope of flow loops with a focus on particle transport. Physics of Fluids, 34(8), 081301.
